# Black TiO_2_ Synthesis by Chemical Reduction Methods for Photocatalysis Applications

**DOI:** 10.3389/fchem.2020.565489

**Published:** 2020-11-17

**Authors:** Luminita Andronic, Alexandru Enesca

**Affiliations:** Department of Product Design, Mechatronics and Environment, Transilvania University of Brasov, Brasov, Romania

**Keywords:** black TiO2, chemical reduction, defect chemistry, photocatalysis, visible light irradiation

## Abstract

Applications of TiO_2_ nanomaterials in photocatalysis, batteries, supercapacitors and solar cells, have seen widespread development in recent decades. Nowadays, black TiO_2_ have won attention due to enhancing the solar light absorption by the formation of oxygen vacancies and Ti^3+^ defects, to promote the separation of photo-generated charge carriers leading to the improvement of the photocatalytic performance in H_2_ production and pollutants degradation. The enhanced photocatalytic activity of black TiO_2_ is also due to a lattice disorder on the surface and the presence of oxygen vacancies, Ti^3+^ ions, Ti-OH and Ti-H groups. Enhancing the optical absorption characteristics of TiO_2_ and change of energy level and band-gap of materials have been successfully demonstrated to improve their photocatalytic activities, especially for black TiO_2_ nanoparticles, which promote visible light absorption. The current review focuses on the investigation of the chemical reduction synthetic route for black TiO_2_ nanomaterials, and their proposed association with green applications such as photodegradation of organic pollutants and photocatalytic water splitting. The synthesis methods of black TiO_2_ involves the changes from Ti^4+^ to Ti^3+^ state, into different strategies: (1) The use of highly active hydrogen species such as H_2_, H_2_/Ar or H_2_/N_2_ gases, and metal hydrides (NaBH_4_, CaH_2_), (2) the reduction by active metals such as aluminum, magnesium and zinc, and (3) organic molecules such as imidazole and ascorbic acid.

## Theoretical Consideration of Black TiO_2_

Chen et al. first reported black TiO_2_ with a narrowed band-gap of 1.5 eV to expand the full spectrum sunlight absorption and promote an increase in the photocatalytic activity, by introducing surface disorders in the TiO_2_ (Chen et al., 2011). Hu et al. observed in 2012 a remarkable enhancement in the visible-light absorption and the photocatalysis of TiO_2_ after hydrogen treatment, attributed to surface disorder and the formation of oxygen vacancies (Hu, 2012; Wang and Chou, 2016; Zhu et al., 2016).

In the past decade, a considerable effort has been committed to preparing black TiO_2_ by introducing Ti^3+^ defects and oxygen vacancies into the titanium oxide lattice (Di Valentin et al., 2009; Su et al., 2014; Li et al., 2015; Tian et al., 2015; Xin et al., 2015). Oxygen vacancy and Ti^3+^ defects are more detectable in black TiO_2_ compared with white TiO_2_. Oxygen vacancies have been detected by a few techniques (Zhang and Park, 2017): electron paramagnetic resonance (EPR), electron spin resonance (ESR), and Raman spectroscopy. Ti^3+^ defects are not proved in white TiO_2_, but they are detected in black TiO_2_ based on X-ray photoelectron spectroscopy (XPS), by EPR (Jedsukontorn et al., 2017) or ESR spectroscopy (Tian et al., 2015). The yellow TiO_2_ synthesis at a low temperature had more oxygen vacancies and Ti^3+^ defects compared with white TiO_2_, which decreases the band-gap from 3.1 to 2.9 eV (Bi et al., 2020).

The formation defects in titanium oxide are given below (Jayashree and Ashokkumar, 2018):

Oxygen vacancy (Kröger-Vink notation $V_{O}^{\cdot \cdot }$) formation at a low oxygen pressure

(1) $$\begin{array}{l} O_{O}↔V_{O}^{\cdot \cdot }+2e^{\prime }+1/2O_{2} \end{array}$$

Titanium interstitials Ti^3+^ (Kröger-Vink notation $Ti_{i}^{\cdot \cdot \;\cdot }$):

(2) $$\begin{array}{l} 2O_{O}+Ti_{Ti}↔Ti_{i}^{\cdot \cdot \cdot }+3e^{\prime }+O_{2} \end{array}$$

Cui et al. (2014) described the generation of the Ti^3+^ and oxygen vacancies, by the equation of the defect (equation 3). They observed that the number of oxygen vacancies is half of the Ti^3+^ sites (equation 3). The absorption increases with the density of Ti^3+^ or O vacancies, as the density of Ti^3+^ increases with the Al reduction in temperature.

(3) $$\begin{array}{l} {2Ti}_{Ti}^{x}+O_{O}^{x}\to {2Ti}_{Ti}^{\prime }+1/2O_{2}+V_{O}^{\cdot \cdot } \end{array}$$

The colorful TiO_2_ with better absorption properties and improved photocatalytic activities compared with white TiO_2_ have been designed through (i) metal doping in which metal replaces Ti^4+^ ions in the TiO_2_ lattice (Chen et al., 2015), (ii) non-metal doping to replace O^2−^ ions in the TiO_2_ lattice (Hamilton et al., 2014), (iii) to replace partial Ti^4+^ and O_2_ ions in the TiO_2_ lattice. To replace Ti^4+^ in TiO_2_ with any cation is more accessible than to substitute O^2−^ with anions (such as nitrogen, carbon, sulfur) due to the difference in the charge state and ionic radii (Lee et al., 2014).

The structural modifications in black titanium oxide, involving Ti^3+^ centers and oxygen vacancies, conduct significant changes in crystallinity, and optoelectronic as well as the surface properties, and the most marked effect is the color changing. Increasing the optical absorption properties and diminishing electron-hole recombination of TiO_2_ are expected to be meaningful for excellent photoactivity.

The optical band gaps of white anatase and rutile TiO_2_ are reported as 3.2 and 3.0 eV, respectively (Haider et al., 2019), that means TiO_2_ can adsorb only the UV part of the solar spectrum. A significant reduction in the TiO_2_ band-gap around 1.23 eV and optical absorption near 1,000 nm in the near-infrared region (Ullattil et al., 2018), has been reported by hydrogenation of anatase nanocrystals under pressure resulting in the black TiO_2_ materials (Chen et al., 2011; Liu et al., 2013).

**Figure 1:**
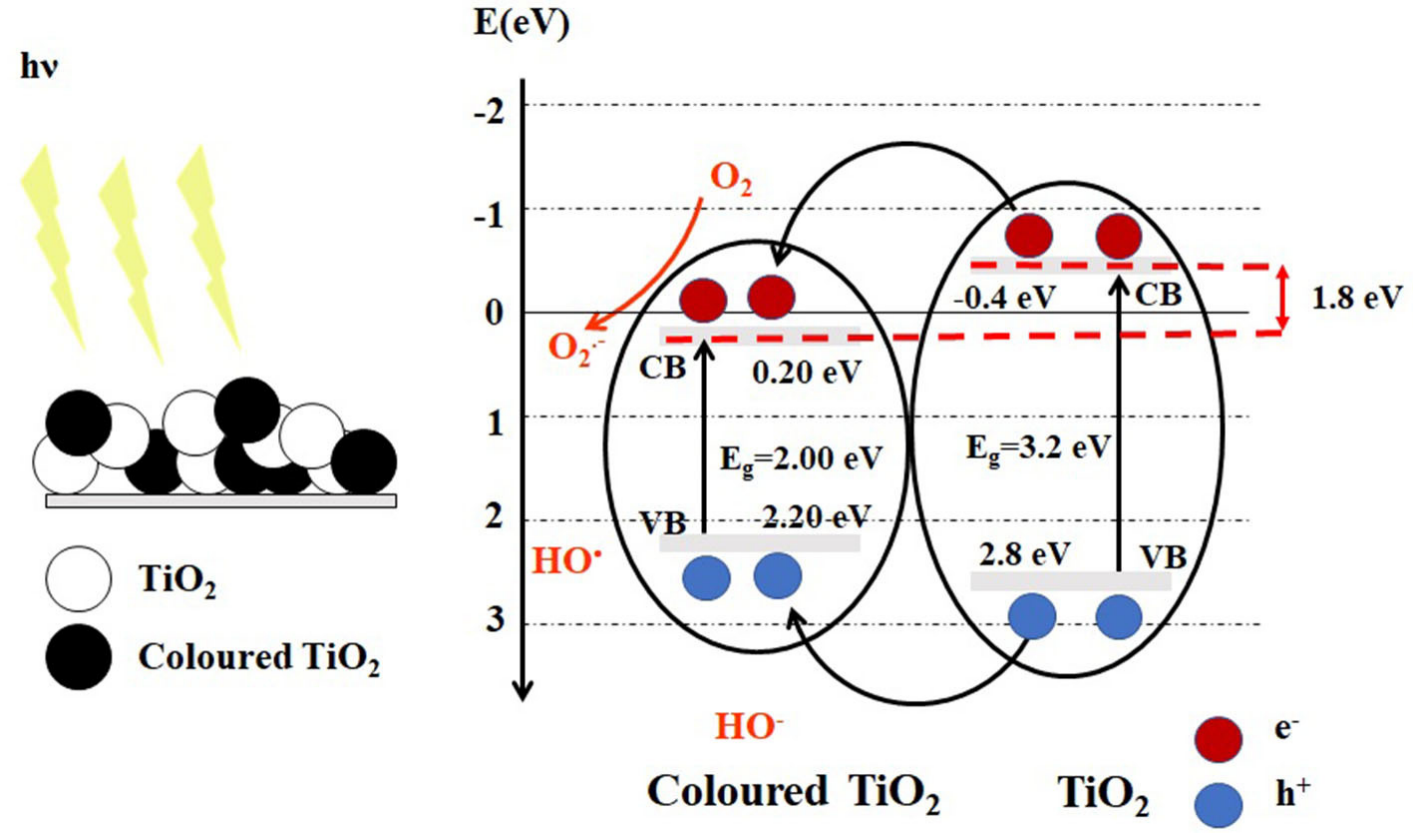
The heterojunction between white TiO_2_ and colored TiO_2_.

The color change of titanium oxide from gray, blue, brown or black color, reflects in turn the optical properties and structural changes (Yan et al., 2017). By exposure to visible light, a heterojunction type I (Isac et al., 2019) is formed between white TiO_2_ and colored TiO_2_, the band energy levels of colored TiO_2_ are included in that of the white TiO_2_, and both heterojunction semiconductors could be excited to produce electrons and holes (Figure 1).

The colored TiO_2_ shows a light absorption around 2eV, by introducing oxygen vacancies ($V_{O}^{\cdot \cdot }$) and Ti^3+^ formation into TiO_2_ lattice (Naldoni et al., 2019) or introducing disordered layers in the surface of crystalline TiO_2_ (Song et al., 2017) enhanced solar light adsorption and served to prove their photocatalytic performance. The oxygen vacancy can significantly affect the electric and optical properties of the materials, by forming a donor level below the conduction band, located at 1.8eV below conduction band of titania as shown in Figure 1. The Ti^3+^ defect is responsible for changes in the electronic conductivity and optical properties. The Ti^3+^ and $V_{O}^{\cdot \cdot }$ defects can be created by the reduction of TiO_2_, either electrochemically, or through gas annealing and exposure in a vacuum (Lee et al., 2014).

Hu emphasize the importance of the crystalline phase of titanium oxide in the synthesis of black TiO_2_; the rutile phase is theoretically easier to obtain than anatase crystalline phase because the formation energy of an oxygen vacancy in the rutile surface (110) is lower (5.5eV) than in the anatase surface (001) (7.54 eV) (Hu, 2012). The most common phase of the black TiO_2_ is rutile or anatase; the rutile phase is formed at a temperature below 500°C. The oxygen deficiency and amorphous surface of TiO_2−x_ were also reported by Tan et al. (2014). The photocatalytic behavior of TiO_2_ was found to depend on the crystalline phase; the white TiO_2_ anatase phase has been shown to have higher photocatalytic efficiency than rutile TiO_2_. Contrary to the previous statement, black rutile TiO_2_ has been reported to have the best photocatalytic performance.

The synthesis methods of black TiO_2_ are significantly affected by the structural, morphological and optical properties; involving the changes from Ti^4+^ to Ti^3+^ state, into different strategies, (i) the use of highly active hydrogen species such as H_2_, H_2_/Ar or H_2_/N_2_ gases, and metal hydrides (NaBH_4_, CaH_2_), (ii) the reduction by active metals such aluminum, magnesium and zinc, (iii) organic molecules such as imidazole and ascorbic acid, have been confirmed to be capable of reducing white TiO_2_ to black titania.

## Synthesis Approach of Black TiO_2_ by Chemical Reduction

The synthesis methods explored through hydrogenation, plasma, chemical reduction, electrochemical reduction, laser ablation in liquid, and oxidation approaches were available in the literature over the last decade for black TiO_2_ photocatalytic materials (Rajaraman et al., 2020).

The synthesis route influences the physicochemical properties and photocatalytic performance of black TiO_2_. A significant number of studies highlight the formation of black TiO_2_ by hydrogen thermal treatment when the samples had surface and bulk defects comparing with plasma treatment under Ar (95%)/H_2_ (5%) atmosphere where the bulk defects were revealed (Wang and Chou, 2016). The color of the samples turned brown at 400°C, while the samples turned black at 500°C. The white TiO_2_ Degussa powder was unchanged under hydrogenation, emphasized the role of precursors and synthesis route (Leshuk et al., 2013).

The reduction strategy can be generally explained in Equation 4, where Red represents the reductant:

(4) $$\begin{array}{l} TiO_{2}+Red\to TiO_{2-x}+RedO_{x} \end{array}$$

The noble gas atmosphere has been considered as reductant due to defective TiO_2−x_ formation in argon, nitrogen atmosphere, and the disordered layer forms only if crystallization is performed in an oxygen-free environment (Tian et al., 2015).

(5) $$\begin{array}{l} TiO_{2}↔TiO_{2-x}+x/2O_{2} \end{array}$$

The first synthesis of black titanium oxide consists of anatase nanoparticles through treating white TiO_2_ nanoparticles (precursors, titanium tetra-isopropoxide, ethanol, hydrochloric acid, deionised water, and Pluronic F127 as an organic template) under a 20.0-bar H_2_ atmosphere at about 200°C for 5 days (Chen et al., 2011).

### NaBH_4_ Reduction

By reducing hydrides, black titanium oxide can be obtained through three approaches: (i) physical mixing of white TiO_2_ and hydride, followed by annealing in an inert atmosphere (Ar, N_2_) at temperature of 300…400°C, (ii) hydrothermal synthesis (Ren et al., 2015) and (iii) sol-gel process (Fang et al., 2014).

The sodium borohydride (NaBH_4_) is a commonly used reducing reagent (Table 1), due to its ability to reduce Ti^4+^ to Ti^3+^, as in Equations (6) and (7), and to produce *in situ* active H_2_ at room temperature, that reduces the white TiO_2_ into black TiO_2_ (Equation 8). During the NaBH_4_ reduction process, boron oxide species are produced due to their insolubility in the ethanol, and it can be easily washed out by HCl solution to remove the surface impurities and expose the color centers on the surface of the catalyst, significantly increasing the visible light absorption. The degradation efficiency increases 9 times after washing with HCl solution (Fang et al., 2014).

(6) $$\begin{array}{l} NaBH_{4}+8OH^{-}\to NaBO_{2}+8e^{-}+6H_{2}O \end{array}$$

(7) $$\begin{array}{l} Ti^{4+}+e^{-}\to Ti^{3+} \end{array}$$

(8) $$\begin{array}{l} BH_{4}^{-}+2H_{2}O\to BO_{2}^{-}+4H_{2} \end{array}$$

**Table 1:** The chemical reduction synthesis methods, properties and photocatalytic applications of representative black TiO_2_ materials.

**Materials**		**Applications**		**References**
**Synthesis**	**Characterization**	**Experimental**	**Photocatalysis efficiency**	
**NaBH**_**4**_ **reduction**				
*TiO_2_ sol-gel synthesis:* Solution A: 5 mL tetrabutyl titanate and 25 mL EtOH. Solution B: 4 mL HNO_3_ (0.6 M) and 5 mL EtOH *NaBH_4_ reduction:* NaBH_4_ add in the sol 0.025, 0.05, 0.1, 0.3 and 0.4 g	*Phase:* anatase, D= 9…35 nm *Band-gap energy:* 2.87 eV *BET surface area:* 18…27 m^2^/g for black TiO_2_ (priscine: 2 m^2^/g)	Rhodamine B 10 mg/L Catalyst 1g/L *Light source:* 500 W tungsten halogen lamp, filter (λ > 420 nm), 300 W high-pressure Hg lamp, filter (λ <365 nm)	*Degradation efficiency:* 100% after 5 h (sample 0.1-TiO_2_). The degradation rate increase 9 time after washing with HCl.	Fang et al., 2014
*TiO_2_ synthesis:* anodic oxidation of titanium foils, annealed *NaBH_4_ reduction:* nanotube arrays were dipped in 0.1 M NaBH_4_ for different times at room temperature	*Phase:* anatase, rutile and brookite *Morphology:* nanotube ~7 mm, pore diameter ~ 100 nm *Band gap energy:* 2.46 eV, (pristine 3.09 eV)	TiO_2_ (working electrodes), Pt (counter-electrode), Ag/AgCl (reference electrode). *Light source:* 300 W Xe lamp, UV cut-off filter of 420 nm, light intensity 0.37 W cm^−2^	*H_2_ production rate:* 1.31% at 0.40 V_RHE_ after 40 min (pristine 0.32% at 0.48 V_RHE_)	Kang et al., 2013
*TiO_2_@TiO_2−*x*_ synthesis:* 4.0 g of Degussa TiO_2_ powder, 1.5 g of NaBH_4_, heated to 300–400°C, Ar, 5–60 min, dark blue TiO_2_ 300°C/50 min	*Phase:* Anatase, rutile *Morphology:* core-shell *Band-gap energy:* 1.1…2.1 eV (priscine 3.1 eV) *BET surface area: 43…50* m^2^/g for black TiO_2_ (priscine: 45 m^2^/g)	Methyl orange 20 ppm (V=50 mL, pH=1), Catalyst 1 g/L	*Degradation efficiency:* 90% after 10 min (Pristine 75%)	Tan et al., 2014
		Methanol (120 mL, 25%), Catalyst 50 mg/1 wt%Pt *Light source:* 300 W Xe lamp, UV cut-off filter of 400 nm	*H_2_ production rate:* 6.5 mmol·h^−1^·g^−1^ (7.2 times better than pristine)	
*TiO_2_ synthesis:* hydrothermal TiCl_4_, ethylene glycol, heated at 150°C/6 h *Defective TiO_2−*x*_ synthesis:* dual-zone tube furnace, Ar, 500°C/1h, TiO_2_:NaBH_4_ mass ratio of 1 to 4	*Phase*: low crystallinity	Methanol (30 mL, 10%) Catalyst 0.03 g/0.03 wt% Rh *Light source:* 500 W mid-pressure Hg lamp and a 420 nm cut-off filter	*H_2_ production rate:* 580 mmol· h^−1^· g^−1^	Xu et al., 2019
**Metal reduction**				
*Reduced TiO_2−*x*_ synthesis:* Aluminum reduction into two zones tube furnace, *p* <0.5 Pa, Al heated at 800°C, and TiO_2_ heated at 300… 600°C, 6 h and 20 h Anneling: 500….900°C, 12h, Ar	*Phase:* anatase, rutile, highly crystalline *Morphology:* core-shell,~25 nm in diameter *Band gap energy:* ~3.2 eV similar to pristine *BET surface area: 42* m^2^/g (priscine: 43 m^2^/g)	Methyl orange 0.1 M (100 mL) Phenol 0.3 M Catalyst 1 g/L	*Degradation efficiecy:* 52% MO (4% pristine) after 6 h 78% Phenol (82% pristine) after 3.5	Wang et al., 2013
		Methanol 25% (120 mL) Catalyst (0.5 wt% Pt) 0.8 g/L *Light source:* UV irradiation: 300 W Hg lamp	*H_2_ production rate:* H_2_ 6.4 mmol h^−1^ g^−1^ (8.5 times higher than that of pristine TiO_2_ (0.75 mmol h^−1^ g^−1^)	
*TiO_2_ hallow sphere synthesis:* 1 mmol tetrabutyl titanate, 0.5 g carbon spheres, stirred for 6 h, washed and dried at 90°C for 6 h. Annealing: 400–500°C, 4 h. *Black TiO_2_*: Aluminum two-zones tube furnace, TiO_2_ 500°C zone, Al 800°C zone. Annealing: 6 h	*Phase:* high crystalline, D~8 nm anatase, rutile (>500°C) *Morphology:* Hollow sphere *Band gap energy: BET surface area:* 168.8 m^2^ g^−1^	Methanol 10% (100 mL) Catalyst: 0.2 g/L *Light source:* 300 W Xe-lamp band-pass filter (λ = 365 nm)	*H_2_ production rate:* 56.7 mmol·h^−1^·g^−1^, 2.5 times higher than pristine	Song et al., 2017
*Reduced TiO_*x*_* (*x* <2) nanoparticles (white, gray, blue, and black) were prepared by reducing P25 TiO_2_ (400 mg) with Mg (60…400 mg). Annealing: 600°C, 4 h, Ar.	*Phase:* TiO_0.89_, TiO_2_ anatase TiO_2_ rutile, D ≈24 nm (as pristine) *Morphology:* core-shell	*Light source:* solar-simulated light irradiance at an intensity of 1000 W m^−2^ (1 Sun).	*Solar thermal conversion efficiency*: Black TiO_x_: 50%	Ye et al., 2017
**Organic molecules reduction**				
*TiO_2−*x*_ hydrothermal synthesis:* L-ascorbic acid (0, 0.3 g and 0.7 g), 70 mL DI water, 3.1 mL of TiCl_3_, NaOH solution (1 mol/L) to pH=4. The mixture was transferred to a 100 mL Teflon- lined stainless steel autoclave and heated at 180°C for 12 h.	*Phase:* Anatase D=10…50 nm *Morphology:* core 10…50 nm *Band gap energy:* 1.0 eV *BET surface area: 64.56 (white), 188.75 (brown), 263.95 m^2^ g^−1^ (black), respectively*	Methylene blue (MB) 20 mg/L (V=40 mL) Phenol 10 mg/L Photocatalyst 0.5 g/L *Light source:* 300 W Xenon lamp, UV cut-off filter (λ > 420 nm)	*Degradation efficiecy:* MB 90% (black TiO_2−x_), 70% (brown TiO_2−x_), 50% (white TiO_2−x_), 5% (pristine), after 100 min Phenol 100% (black TiO_2−x_), after 80 min	Wajid Shah et al., 2015

The rate of the generation of H_2_ is higher in acidic conditions at experiments performed at 25°C with 20 g water per gram of sodium borohydride, and a wt. ratio accelerators/NaBH_4_, around 1. The representative acidic materials that act as accelerators are tartaric acid, citric acid, succinic acid, oxalic acid (85…98% hydrogen liberated after 3 min), ammonium carbonate, maleic acid, aluminum sulfate, sodium diacid phosphate (90….80% of H_2_ after 10 min), maleic anhydride, ammonium chloride, benzoic acid (80….65% of H_2_ after 10 min). The catalytic effect of metals salt (cobalt, aluminum) was also demonstrated (Schlesinger et al., 1953).

The treatment of the TiO_2_ nanotube in NaBH_4_ for a short time (20–40 min) reduced the surface of TiO_2_ into Ti^3+^, and introduced an oxygen vacancy that creates localized states, producing a narrower band-gap of 2.46 eV, which extends its optical absorption to the visible region comparing with 3.09 eV for pristine TiO_2_ nanotubes (Table 1) (Kang et al., 2013). The films show good stability and excellent reproducibility of the samples.

Tan et al. report a solid-state chemical reduction of TiO_2_ at mild temperatures (300–350°C), for different times up to 1 h, an approach for large-scale production for visible light photocatalysis and solar-driven H_2_ production (Table 1). The preparation of black TiO_2_ followed the procedure: 4 g of TiO_2_ Degussa P25 powder was mixed at room temperature with 1.5 g of NaBH_4_ (98%) and heated in a tubular furnace under Ar atmosphere, up to 300°C and held for 5–120 min. When the temperature increase to 350°C, the black titanium oxide was obtained in 60 min. The colored powders from light blue to black were washed with deionised water and ethanol several times to remove unreacted NaBH_4_ and dried at 70°C (Tan et al., 2014).

Xu et al. obtained black TiO_2_ powders in a dual-zone quart tube furnace using titanium oxide synthesized of TiCl_4_ and ethylene glycol at 150°C for 6 h in a Teflon-lined stainless-steel autoclave with NaBH_4_ as reductant agent (Table 1). The reduction was carried out in an argon atmosphere, between 200 and 500°C for 1 h. The presence of Ti^3+^ and oxygen vacancy defects significantly increased the intensity of the band absorption in the visible spectrum range (Xu et al., 2019).

### Metal Reduction

In recent studies, active metals such as magnesium, lithium, aluminum and zinc were used for the synthesis of black TiO_2_ with oxygen-deficient metal oxides (Zu et al., 2019).

Ou et al. developed a room-temperature lithium reduction strategy removing oxygen, and generating oxygen vacancies into the titanium dioxide nanoparticles lattice. Lithium metal with a high reductive capacity can reduce a significant number of metal oxides at room temperature (TiO_2_, ZnO, SnO_2_, CeO_2_). TiO_2_ Degussa P25 and lithium powders (0.5% wt%) were mixed with a dispersant (dimethyl carbonate), then washed with diluted hydrochloric acid to remove lithium oxide, centrifugate and washing. The dried powders appear in different colors ranging from blue to black, and shift with the increase in lithium content (Ou et al., 2018).

The aluminum reduction of titanium oxide produces black TiO_2_ in two ways: (i) reduction approach in an evacuated two-zone vacuum furnace, low temperature (300–600°C) for TiO_2_ and high temperature (800°C) for aluminum and (ii) thermal treatment of a mixture of TiO_2_ and aluminum powder (Table 1) (Wang et al., 2013). The black TiO_2_ nanotube arrays have been used as a photoanode of photoelectrochemical cells for water-splitting, which was about 5 times higher than that of pristine (Cui et al., 2014).

Song et al. Herein prepared the black TiO_2_ nanoparticles through subsequent Al reduction, with hollow nanosphere morphology, high crystallinity, small grain size (~8 nm), and high surface area (168.8 m^2^·g^−1^) for photocatalytic hydrogen generation (56.7 mmol h^−1^·g^−1^) 2.5 times higher than pristine TiO_2_ nanostructures. The aluminum reaction was performed for 6 h in an evacuated two-zone furnace, pristine TiO_2_ hollow nanospheres were placed in the low-temperature zone (400….600°C), and the aluminum powder was placed at 800°C (Table 1) (Song et al., 2017).

Sinhamahapatra et al. report in 2015 a magnesiothermic reduction under a 5% H_2_/Ar atmosphere followed by acid treatment to synthesize reduced black TiO_2_ nanoparticles with improved optical absorption in the visible and infrared region for enhanced photocatalytic hydrogen production in the methanol-water system in the presence of Pt as a co-catalyst (Sinhamahapatra et al., 2015).

Nanoparticles with different colors were synthesized by Ye et al. (2017) using Mg as a reductant (Table 1). Commercial P25 TiO_2_ nanoparticles were mixed with Mg powder into wt. ratio 20:3, 10:3, 5:3, and 1:1, before being purged with argon for 15 min and calcined at 600°C under an Ar atmosphere for 4 h. The TiO_x_ (x <2) nanoparticles with different Ti/O ratios increased with the increasing addition of Mg in the reaction (Equation 9) and colors (turned gray, blue-gray, light black, and dark black, respectively). The nanoparticles are material for converting solar energy to the thermal energy for evaporation of water.

(9) $$\begin{array}{l} TiO_{2}+\left(2-x\right)Mg\to TiO_{x}+\left(2-x\right)MgO \end{array}$$

### Organic Molecules Reductant

Seok et al. synthesis Ti^3+^ self-doped TiO_2_ using the sol-gel route: 5 g of TiOSO_4_, 250 ml distilled water, 1.5 g urea as a dispersant, NaOH was added (pH=7), and precursors annealed under an oxidative atmosphere at 350°C for 6 h in the presence of 2-methylimidazole and HCl when the Ti^4+^ was reduced to Ti^3+^ which resulted in lower internal resistance and improved electronic conductivity with application in Li-ion batteries as anode materials with a capacity retention of 88% at 50°C (Seok et al., 2016).

A facile hydrothermal approach, described in Table 1, has been developed by Wajid et al. to prepare defective TiO_2−x_ high surface nanocore using ascorbic acid as a reductant, established theoxygen vacancy concentration and tunable band-gap by setting the amount of ascorbic acid (Wajid Shah et al., 2015).

The synthesis methods of black TiO_2_ changed the phase and crystallinity, morphology, band-gap and BET surface area, essential elements in photocatalysis as described in Table 1. The experimental conditions (pollutants and catalysts concentration, light irradiation and intensity) influence the pollutant degradation efficiency and H_2_ production rate by photocatalysis (Table 1).

The synthesis techniques to obtain black TiO_2_ and defective TiO_2−x_ follow four strategies: introducing surface disorders, Ti^3+^ defects, oxygen vacancies, Ti-OH and Ti-H groups to narrowing the band-gap for photo-related applications (Liu et al., 2017; Yan et al., 2017).

Chemical reduction is associated with a change in the oxidation state of Ti^4+^ with the formation of Ti^3+^ species responsible for the electronic conductivity, essential for many applications of TiO_2_, especially photocatalysis (Di Valentin et al., 2009). The surface Ti^3+^ species are unstable and can be quickly oxidized by oxygen in air or water, developing a method to synthesize black TiO_2_ materials that with improved visible-light photocatalytic activity is a challenge (Zheng et al., 2013).

## A Fundamental Process in Photocatalytic Activity of Black TiO_2_

In the past years, black titanium oxide has attracted attention in different fields, such as photocatalytic pollutants degradation (Chen et al., 2011; Li et al., 2019; Plodinec et al., 2019), photocatalytic hydrogen production through water splitting (Wang et al., 2017; Pan et al., 2019), photocatalytic CO_2_ reduction (Qingli et al., 2015; Zhao et al., 2016; Gao et al., 2020), solar–thermal material (Ye et al., 2017), supercapacitor (Zhi et al., 2016; Huang et al., 2018), photoanode in Dye-Sensitized Solar Cells (Ullattil et al., 2017), Lithium-ion batteries (Kim et al., 2017) (Yang et al., 2018), and medicine (Ni et al., 2017; Mazare et al., 2019).

The principle of the semiconductors photocatalysis consists of the following components: photon absorption, carriers separation, carrier diffusion simultaneously with carrier transport, catalytic efficiency and mass transfer of reactants and products (Takanabe, 2017).

*Photon absorption*: if the semiconductor has energy equal to or greater than E_g_ and consequent excitation of electrons (e^−^) to the CB leaving positively charged vacancies, holes (h^+^), in the VB (Figure 1)
*Carriers separation:* the heterojunction between nanoparticles can better band gap arrangement, to improve the separation of photo-generated charge carriers (Figure 1), which is advantageous of improving the photocatalytic performance. The oxygen vacancy defects and Ti^3+^ centers on the surface of TiO_2_ favor the separation of charge carriers (electrons and holes) and can trap the hole.
*Carrier diffusion simultaneously with carrier transport*. The photo-generated electrons can initiate the reduction processes, including O_2_ reduction to superoxides, H_2_ generation, and CO_2_ reduction to methane, methanol, or formaldehyde (Wen et al., 2015). The electron transfer is significant for the knowledge of the fundamental concepts of photocatalytic processes and to have an opinion about design and industrialization of the photocatalytic process (Mohamed and Bahnemann, 2012).
*The transfer of electron/hole pairs to the interface initiates the redox reaction*. The lifetime of the photo-generated charge carrier determines the efficiency of photocatalytic processes (Takanabe, 2017). Hence, increasing the efficiency of charge separation/transport in semiconductor nanoparticles is one of the major problems in photocatalysis to be addressed by the black TiO_2_.
The presence of oxygen vacancies in TiO_2_ can efficiently *extend the visible light absorption range of titania* because the localized oxygen vacancy states are located at 0.75 to 1.18 eV below the conduction band of TiO_2_ (Asahi et al., 2001) (Figure 1). The hydroxyl radicals (HO∙) can be formed when hydroxyl anions (HO^−^), and adsorbed water trap the holes, which are capable of degrading the organic pollutants in wastewater.

## Summary and Outlook

The current review focuses on the investigation of the chemical reduction synthetic route for black TiO_2_ nanomaterials, and their applications related to the environmental application such as photodegradation of organic pollutants and photocatalytic water splitting.

Since 1972, when Fujishima and Honda (Fujishima and Honda, 1972) reported about the water-splitting process using a TiO_2_ electrode under UV irradiation, photocatalysis has attracted attention. The solar-driven applications of TiO_2_ have been limited due to its band-gap (around 3.2 eV). A remarkable step in solar-driven photocatalysis was presented in 2011 by Chen and co-authors when black TiO_2_ enhanced the photocatalytic activity of TiO_2_. In the last years, many studies have focused on the synthesis and explanation of different properties of black-TiO_2_ to improve the activity of the photocatalyst under visible irradiation. An important drawback is the synthesis requirements such as long annealing treatments (a few days), and the high pressure of hydrogen atmosphere, up to 20 bar.

The colored TiO_2_ can turn from white to yellow, blue, brown or black, due to the change in optical properties (modification of its band-gap), and defects in the surface layers that enhanced solar light adsorption and photocatalytic reactions. Among the colored forms, black TiO_2_ has been one of the most investigated because it can get excellent optical, chemical and electronic properties due to at least one of these characteristics: the presence of Ti^3+^ ions, oxygen vacancies undetectable in white TiO_2_ and usually present in black TiO_2_, structural disorder/defects in the surface, Ti-OH groups, Ti-H groups, and modifications of the valence band edge. The colored TiO_2_ has rich oxygen vacancies and Ti^3+^ defects, which conduct to better conductivity for electron transfer, increased visible absorption and higher conduction band potential. The oxygen vacancies and Ti^3+^ defects can act as traps for reducing the recombination of e^−^/h^+^ pairs and enhancing the photocatalytic activity.

The chemical reduction methods include the reduction of TiO_2_ with active hydrogen species such as H_2_, H_2_/Ar or H_2_/N_2_ gases, using high temperatures with active metals such aluminum, magnesium and zinc powders, or the reduction of TiO_2_ in solution with NaBH_4_ and organic molecules such as imidazole and ascorbic acid can effectively lead to the color change of TiO_2_ into black color. The color change of TiO_2_ depends on the synthesis conditions, such as pressure, temperature, time, and the reducing agent, featuring different structural (lattice changes or disordering), chemical (formation of Ti^3+^, oxygen vacancies, Ti-H, Ti-OH), physical properties (such as optical properties), and photocatalytic activities in both hydrogen generation and organic pollutant removal. The thermal treatment changes the color of the samples between yellow at 300–350°C, brown at 400°C and black above 450°C, the crystal structure has no major changes due to hydrogenation.

Black titanium oxide is a versatile photocatalyst with an extended absorption spectrum into the vis light range of the solar spectrum. From both a material and a chemical reaction perspective, this may provide new opportunities in efficiently utilizing the visible-light region of the spectrum to finally improve the efficiency of black TiO_2_ nanomaterials for practical photocatalytic applications.

## Author Contributions

LA planned the content and wrote the manuscript. AE contributed to the photocatalysis chapter. All authors contributed to the article and approved the submitted version.

## Conflict of Interest

The authors declare that the research was conducted in the absence of any commercial or financial relationships that could be construed as a potential conflict of interest.
